# Isolated Salter-Harris Type II Fracture of the Distal Ulna

**DOI:** 10.7759/cureus.15552

**Published:** 2021-06-09

**Authors:** Vasileios Mitrousias, Vasileios Amprazis, Christos Baltas, Theofilos Karachalios

**Affiliations:** 1 Orthopaedic Department, General University Hospital of Larissa, Larissa, GRC

**Keywords:** distal forearm fracture, isolated ulnar fracture, epiphyseal fracture, salter-harris ii ulnar fracture, growth disturbance

## Abstract

Isolated distal ulna epiphyseal plate injuries are very rare and are often associated with early epiphyseal plate arrest. A 13-year-old boy sustained an isolated minimally displaced Salter-Harris type II fracture of the left distal ulna following a fall from a bicycle. The fracture was reduced, and a long arm plaster cast was applied for four weeks. At the six-month follow-up, the patient presented with a painless, full range of movement of the left wrist, but on radiological examination, a mild shortening of the ulna was detected. We plan to regularly evaluate this patient until distal epiphyseal plate closure and surgically intervene if necessary. To our knowledge, this is the third Salter-Harris type II distal ulnar fracture ever reported, and the second treated nonoperatively. It was shown to be associated with a mild growth disturbance. Although Salter-Harris type II injuries are considered benign, surgeons should closely evaluate this rare type II isolated distal ulnar fracture and inform parents regarding possible future complications, which range from clinically insignificant cosmetic deformity to severe instability of the distal radioulnar joint, depending on the degree of shortening.

## Introduction

Fractures of the distal forearm are common in children and adolescents and are usually located in the distal radius [1]. These injuries may be combined with ulnar fractures, but isolated injuries of the distal ulnar epiphyseal plate are rare [1,2]. Despite their rare incidence, recognition and appropriate treatment are crucial to prevent growth disturbances as the distal ulnar epiphyseal plate accounts for 70-80% of the ulnar growth [1,2]. To our knowledge, we present the third-ever reported isolated Salter-Harris type II distal ulnar fracture, complicated by mild early growth disturbance. Additionally, we comment on the need for close observation of this injury pattern and the need to inform parents of the possibility of future complications.

## Case presentation

**Figure 1:**
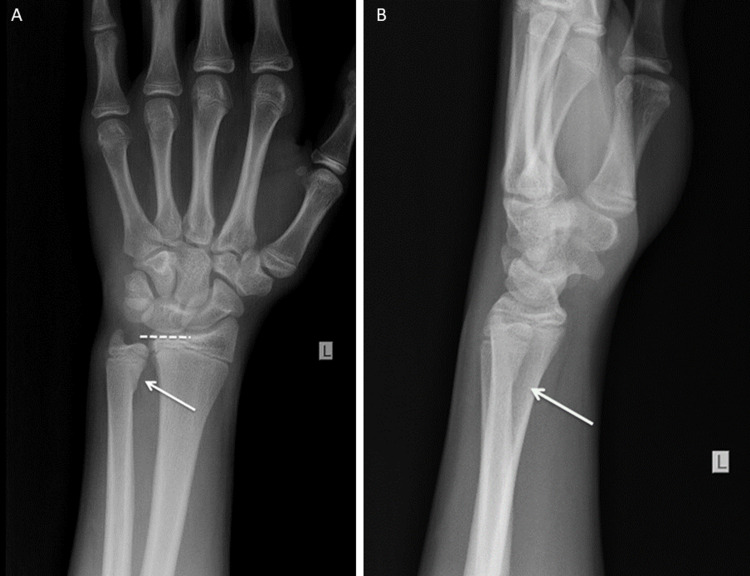
Anteroposterior (A) and lateral (B) radiographs of the injured left wrist showing the epiphyseal plate fracture (arrows).

A 13-year-old boy presented to the emergency department following a fall from a bicycle. He experienced ulnar-sided wrist pain in his left, nondominant hand. He reported landing on his outstretched left hand, with most of the impact being sustained by the ulnar side of the wrist. Physical examination revealed tenderness and mild swelling on the dorsal ulnar side of the wrist, without ecchymosis or gross deformity. Wrist range of motion was slightly limited due to pain. The neurovascular examination was normal. Anteroposterior (Figure 1A) and lateral (Figure 1B) radiographs of the left wrist showed a Salter-Harris type II fracture of the distal ulna.

**Figure 2:**
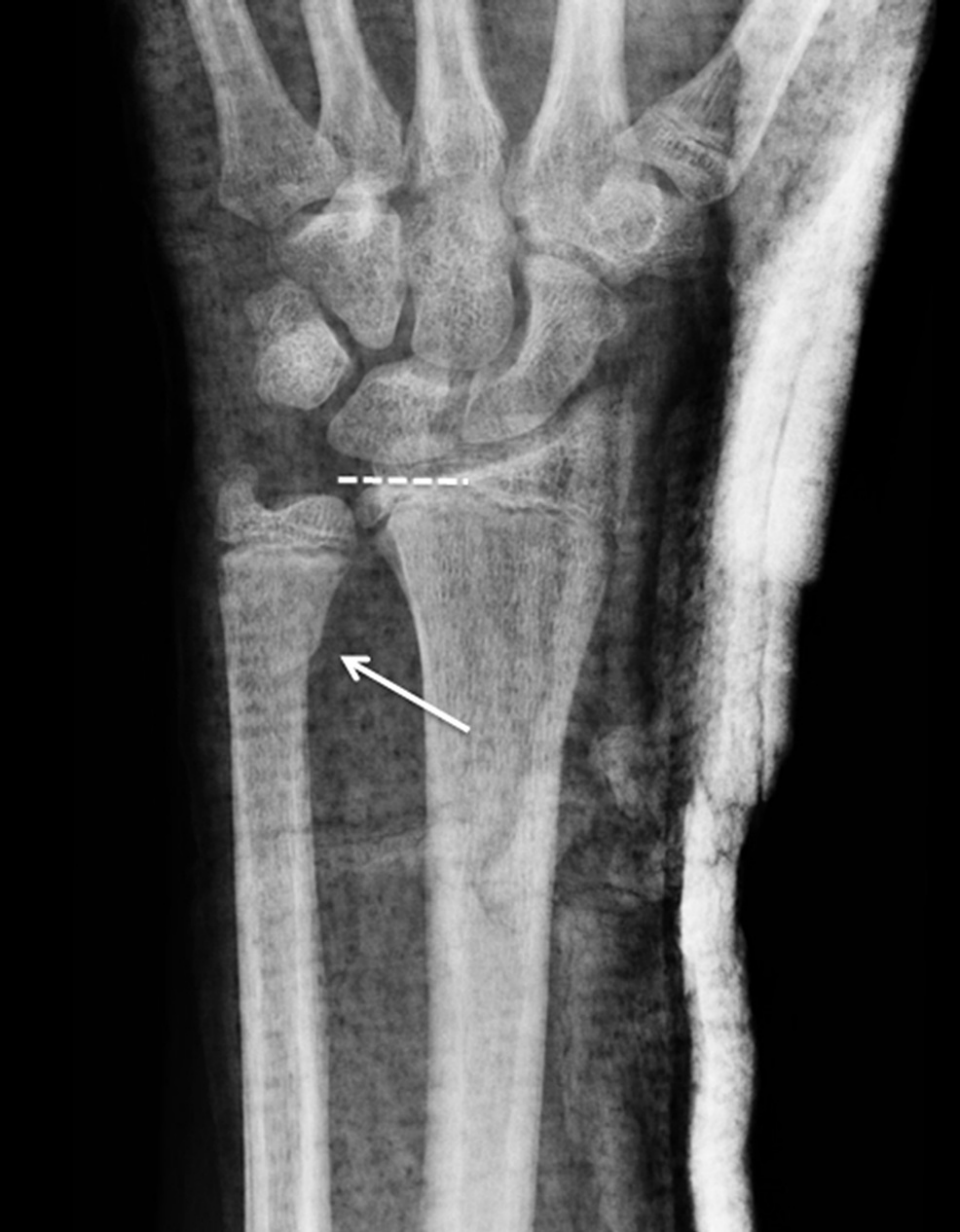
Anteroposterior radiograph of the left wrist following reduction.

The radius was intact. An attempt at reduction was performed, and the wrist joint was immobilized in a long-arm plaster cast. Repeat radiographs were performed one week later (Figure 2).

**Figure 3:**
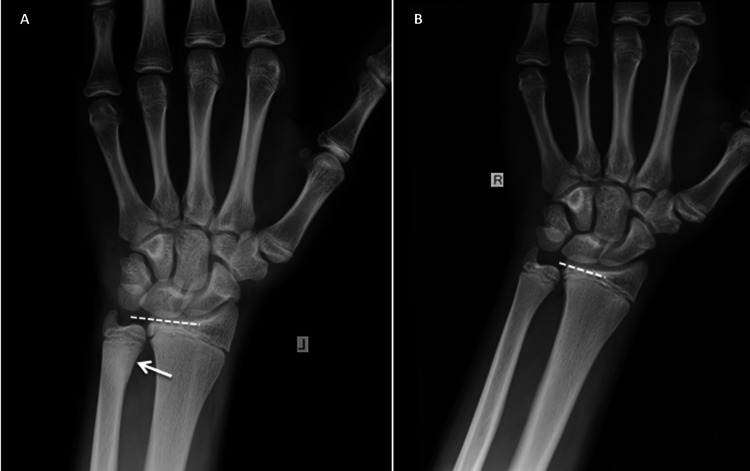
Anteroposterior radiographs of the left (A) and the right (B) wrists at six months following injury.

Following cast removal at four weeks, the patient soon gained a full range of painless wrist movement. On radiological examination at six months, mild shortening of the ulna was recorded (Figure 3). The patient and parents were informed about the possibility of future surgical intervention, and regular (every six months) clinical and radiological follow-up were planned until maturity.

## Discussion

Injuries of the distal radial epiphyseal plate are common in both children and adolescents, but equivalent isolated epiphyseal injuries of the distal ulna are extremely rare [1,2]. Cannata et al., in a long-term, follow-up study, reported five such fractures in combination with distal radial metaphyseal fractures, but only one isolated ulnar injury, in a sample of 163 patients [3]. During our extensive literature research, we were able to identify only five cases, two of which were classified as Salter-Harris type IV, one as type III, and two as type II [4-8] (Table 1). In all five cases, the mechanism of injury was described as a fall on the outstretched hand from either a bike or a motorcycle, which is similar to the presented case. The previously proposed combination of ulnar deviation-dorsiflexion of the wrist in a pronated forearm was also reported by our patient after an extensive inquiry regarding the history of his trauma.

**Table 1:** Clinical details from the few previously published studies.

Authors (year)	Age	Salter-Harris type	Treatment	Premature physeal closure
Marrannes et al. (2020) [4]	14	IV	Open reduction - Kirschner wire fixation	Yes
Yukata et al. (2018) [5]	13	III	Below-the-elbow splint	No
Kasis et al. (2004) [6]	12	IV	Open reduction - Kirschner wire fixation	Yes
Evans et al. (1990) [7]	11	II	Open reduction - Kirschner wire fixation	No
Engber et al. (1986) [8]	15	II	Open reduction - Long-arm splint	Yes

The specific anatomical characteristics of the ulnar side of the wrist are thought to be protective for the ulnar epiphyseal plate. The triangular fibrocartilage complex is believed to act as a cushion between the ulna and the proximal carpal row [6]. Moreover, its wide attachment throughout the entire length of the ulnar styloid process propagates the traumatic deforming forces directly to the styloid process, rather than the epiphyseal plate [9]. This wide, powerful attachment is also considered to be the cause of the avulsion of the ulnar styloid process in concomitant fractures of the distal radius, explaining why dislocated radial fractures are usually accompanied by fractures of the ulnar styloid process [9].

Epiphyseal plate fractures are usually classified using the Salter-Harris criteria [10]. In general, type I and II fractures are considered benign injuries, are usually managed with closed reduction and immobilization, and premature epiphyseal plate arrest and growth disturbances are rare [1,11,12]. Type III and IV fractures are more complex and may require open reduction and internal fixation using Kirschner wires [1,11,12]. However, in distal ulnar epiphyseal injuries, it seems that an open approach is usually necessary to achieve a satisfying reduction as closed reduction may be impossible due to the interposition of the capsule [8,13] or of the extensor carpi ulnaris tendon [7]. The present case is the second case in the literature managed nonoperatively as the fracture was minimally displaced. Although it was a minimally displaced Salter-Harris type II pattern, it resulted in an early mild growth disturbance.

Salter-Harris classification of epiphyseal plate fractures is not closely correlated with clinical outcome. Other factors such as age, attempts at reduction, and energy of the injury have been proposed as better prognostic factors [14]. In ulnar epiphyseal plate fractures, growth disturbances are frequent. Golz et al. reported premature epiphyseal plate closure and subsequent ulnar shortening in 55% of patients sustaining injuries of the distal ulnar after wrist trauma also involving a fracture of the radius [2]. Zimmermann et al. reported negative ulnar variance in 10% of patients during a long-term study of more than 200 distal forearm fractures [15]. A high percentage of premature epiphyseal plate closure also derives from the analysis of the five published cases as in three of them a negative ulnar variance was observed during follow-up [4,5,8]. As stated before, this complication is basically explained by the fact that the distal physeal plate of the ulna accounts for 70-80% of ulnar growth, so fractures in the area may lead to axial shortening of the ulna. This shortening may be clinically insignificant. In fact, in the study by Zimmermann et al., only one patient complained of pain at the distal radioulnar joint under stress [15]. However, it may cause significant problems during activity. Depending on the degree of shortening, it may simply cause patient dissatisfaction due to a cosmetic deformity, but in more severe cases, it can be clinically manifested as persistent pain, limited range of motion, and/or loss of grip due to compensations observed in the radius or instability of the distal radioulnar joint [4,16]. Thus, long-term follow-up after treatment is important in patients with such fractures to identify complications, which may then be addressed successfully with ulnar distraction or closing wedge osteotomy of the radius depending on the patient’s needs [17].

## Conclusions

Although fractures of the distal forearm are common in children and adolescents, isolated, distal, epiphyseal fractures of the ulna are rare. This is the third-ever reported isolated Salter-Harris type II distal ulnar fracture, complicated by mild early growth disturbance. The present case highlights the need for high suspicion and close observation of this injury pattern and the need to inform parents of the possibility of future complications.

## References

[1] Peterson HA (2007). Epiphyseal growth plate fractures.

[2] Golz RJ, Grogan DP, Greene TL, Belsole RJ, Ogden JA (1991). Distal ulnar physeal injury. J Pediatr Orthop.

[3] Cannata G, De Maio F, Mancini F, Ippolito E (2003). Physeal fractures of the distal radius and ulna: long-term prognosis. J Orthop Trauma.

[4] Marrannes S, Lambrecht D, Decramer A (2020). Reduction of an unusual Salter-Harris type IV fracture of the ulna. Case Rep Orthop.

[5] Yukata K, Nakai S, Ikeda M, Hamawaki JI (2018). Isolated Salter-Harris type III physeal fracture of the distal ulna. J Hand Surg Asian Pac Vol.

[6] Kasis AG, Hekal WEA, Mubashir A (2004). Isolated Salter-Harris type IV fracture of the distal ulna in a 12-year-old boy. Eur J Trauma.

[7] Evans DL, Stauber M, Frykman GK (1990). Irreducible epiphyseal plate fracture of the distal ulna due to interposition of the extensor carpi ulnaris tendon. A case report. Clin Orthop Relat Res.

[8] Engber WD, Keene JS (1985). Irreducible fracture-separation of the distal ulnar epiphysis. Report of a case. J Bone Joint Surg Am.

[9] Ogden JA, Beall JK, Conlogue GJ, Light TR (1981). Radiology of postnatal skeletal development. IV. Distal radius and ulna. Skeletal Radiol.

[10] Salter RB, Harris WR (1963). Injuries involving the epiphyseal plate. J Bone Joint Surg Am.

[11] Peterson CA, Peterson HA (1972). Analysis of the incidence of injuries to the epiphyseal growth plate. J Trauma.

[12] O'Hagan T, Reddy D, Hussain WM, Mangla J, Atanda A Jr, Bielski R (2012). A complex injury of the distal ulnar physis: a case report and brief review of the literature. Am J Orthop (Belle Mead NJ).

[13] Faraj AA, Kumar MS, Ketzer B, Rawes M (2000). An irreducible Salter-Harris type IV distal ulna fracture. Injury.

[14] Lee BS, Esterhai JL Jr, Das M (1984). Fracture of the distal radial epiphysis. Characteristics and surgical treatment of premature, post-traumatic epiphyseal closure. Clin Orthop Relat Res.

[15] Zimmermann R, Gschwentner M, Kralinger F, Arora R, Gabl M, Pechlaner S (2004). Long-term results following pediatric distal forearm fractures. Arch Orthop Trauma Surg.

[16] Andersson JK, Lindau T, Karlsson J, Fridén J (2014). Distal radio-ulnar joint instability in children and adolescents after wrist trauma. J Hand Surg Eur Vol.

[17] Price C (2004). Complications in Orthopaedics Pediatric Upper Extremity Fractures.

